# Viral load of SARS-CoV-2 in surgical smoke in minimally invasive and open surgery: a single-center prospective clinical trial

**DOI:** 10.1038/s41598-023-47058-z

**Published:** 2023-11-20

**Authors:** Amila Cizmic, Vanessa M. Eichel, Niklas M. Weidner, Philipp A. Wise, Felix Müller, Ingmar F. Rompen, Ralf Bartenschlager, Paul Schnitzler, Felix Nickel, Beat P. Müller-Stich

**Affiliations:** 1 Department of General, Visceral and Thoracic Surgery, University Medical Center Hamburg-Eppendorf, Hamburg, Germany; 2 Department of Infectious Diseases, Section Infection Control University Hospital Heidelberg, Heidelberg, Germany; 3 Department of Infectious Diseases, Virology, Heidelberg University, Heidelberg, Germany; 4 Department of Infectious Diseases, Medical Microbiology and Hygiene, University Hospital Heidelberg, Heidelberg, Germany; 5 Department of General, Visceral and Transplantation Surgery, University Hospital Heidelberg, Heidelberg, Germany; 6 Department of Infectious Diseases, Molecular Virology, University Hospital Heidelberg, Heidelberg, Germany; 7 Department of Digestive Surgery, University Digestive Healthcare Center Basel, Kleinriehenstrasse 30, 4058 Basel, Switzerland

**Keywords:** Diseases, Infectious diseases, Viral infection

## Abstract

At the beginning of the COVID-19 pandemic, it was assumed that SARS-CoV-2 could be transmitted through surgical smoke generated by electrocauterization. Minimally invasive surgery (MIS) was targeted due to potentially higher concentrations of the SARS-CoV-2 particles in the pneumoperitoneum. Some surgical societies even recommended open surgery instead of MIS to prevent the potential spread of SARS-CoV-2 from the pneumoperitoneum. This study aimed to detect SARS-CoV-2 in surgical smoke during open and MIS. Patients with SARS-CoV-2 infection who underwent open surgery or MIS at Heidelberg University Hospital were included in the study. A control group of patients without SARS-CoV-2 infection undergoing MIS or open surgery was included for comparison. The trial was approved by the Ethics Committee of Heidelberg University Medical School (S-098/2021). The following samples were collected: nasopharyngeal and intraabdominal swabs, blood, urine, surgical smoke, and air samples from the operating room. An SKC BioSampler was used to sample the surgical smoke from the pneumoperitoneum during MIS and the approximate surgical field during open surgery in 15 ml of sterilized phosphate-buffered saline. An RT-PCR test was performed on all collected samples to detect SARS-CoV-2 viral particles. Twelve patients with proven SARS-CoV-2 infection underwent open abdominal surgery. Two SARS-CoV-2-positive patients underwent an MIS procedure. The control group included 24 patients: 12 underwent open surgery and 12 MIS. One intraabdominal swab in a patient with SARS-CoV-2 infection was positive for SARS-CoV-2. However, during both open surgery and MIS, none of the surgical smoke samples showed any detectable viral particles of SARS-CoV-2. The air samples collected at the end of the surgical procedure showed no viral particles of SARS-CoV-2. Major complications (CD ≥ IIIa) were more often observed in SARS-CoV-2 positive patients (10 vs. 4, *p* = 0.001). This study showed no detectable viral particles of SARS-CoV-2 in surgical smoke sampled during MIS and open surgery. Thus, the discussed risk of transmission of SARS-CoV-2 via surgical smoke could not be confirmed in the present study.

## Introduction

The pandemic of coronavirus disease 2019 (COVID-19) caused by severe acute respiratory syndrome coronavirus 2 (SARS-CoV-2) confronted the healthcare system around the world with numerous challenges, including the risk of airborne transmission in the hospital environment^1–6^. One of the crucial sources of airborne transmission in the hospital environment is the aerosol-generating medical procedure^2,6–8^. Aerosol-generating medical procedures are all procedures that produce airborne particles known as aerosols^9^. Aerosol-generating medical procedures include, amongst others, aerosolization of the blood through irrigation or suction and inducing surgical smoke through electrocauterization in surgical procedures^10,11^.

Several publications have reported the presence of various viral particles in surgical smoke, which increased safety concerns for healthcare workers and patients during the COVID-19 pandemic^12–17^. SARS-CoV-2 particles in the blood, urine, and stool samples have indicated a potential risk of airborne transmission through surgical smoke during both open and minimally invasive surgery (MIS)^18–22^. MIS has mainly been targeted due to the possibly higher concentration of surgical smoke in the pneumoperitoneum than in open surgery^23^. The leading guidelines referring to surgical practice in the era of the COVID-19 pandemic have advised caution when performing MIS procedures due to the unknown risk of the aerosolization of the SARS-CoV-2 particles^24–26^. In some countries, a shift towards an open surgical approach was observed in already standardized MIS procedures^27–29^. Despite increasing awareness of the association between aerosol-generating medical procedures and the airborne transmission of different viruses, there is still a limited understanding and evidence of the risks of specific surgical procedures and SARS-CoV-2 transmission^6,7,10^.

In current literature, SARS-CoV-2 has been detected on filters attached to the trocars during MIS in SARS-CoV-2 infected patients^30^. However, no studies have analyzed the presence and infectivity of SARS-CoV-2 in surgical smoke during open and MIS procedures, which leaves us vastly unprepared for future pandemics regarding preventing airborne transmission in a clinical setting. This study aimed to detect the presence of SARS-CoV-2 in surgical smoke and determine the infectivity and the potential risk of airborne transmission during open and MIS procedures.

## Materials and methods

This prospective, single-center clinical trial has been reported according to the STROBE guidelines^31^. It was conducted between March 2021 and July 2022 at the Department of General, Visceral and Transplantation Surgery, University Hospital Heidelberg, Germany. The trial was conducted in compliance with the Helsinki Declaration and Ethical Guidelines for Medical and Health Research Involving Human Subjects. It has been approved by the Ethics Committee of Medical Faculty University Heidelberg (S-098/2021) and registered in the German Clinical Trials Register (DRKS00025497).

The inclusion criteria for the SARS-CoV-2 group was positive SARS-CoV-2 reverse transcription-polymerase chain reaction (RT-PCR) of nasopharyngeal swabs and the indication for open or MIS procedure. The control group included patients with negative nasopharyngeal SARS-CoV-2 RT-PCR tests undergoing elective open or MIS abdominal procedures. If the patients had multiple nasopharyngeal swabs during the hospital stay, the latest one before surgery was considered for inclusion in the study. Informed consent was obtained from all included patients or their legal guardians.

The following samples were collected in both groups during the surgery: nasopharyngeal and intraabdominal swabs, blood, urine, stool, surgical smoke for 30 min after starting electrocauterization, heat, and moisture (HME) filter membrane from the mechanical ventilation, and air samples from the operating room (OR) for 30 min at the approximate end of surgery.

**Figure 1:**
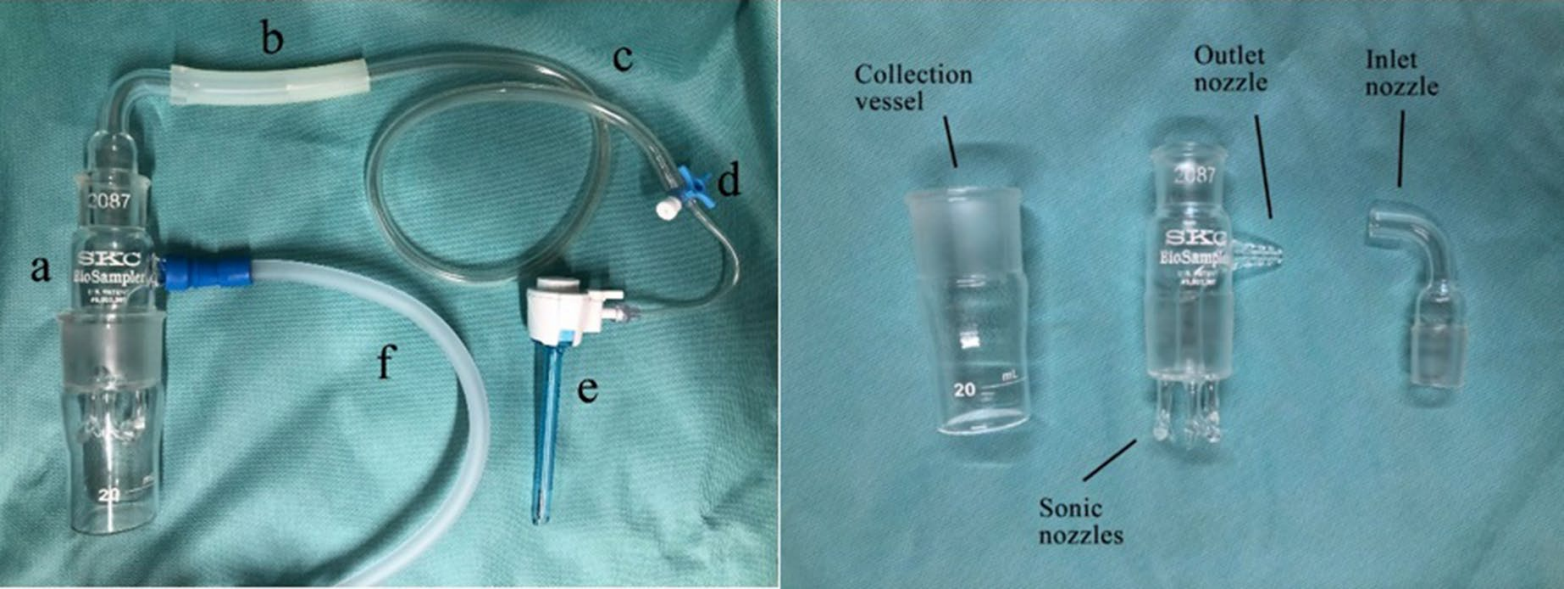
On the left: SKC BioSampler attached to connecting tubes to the patient side and the vacuum pump: a: SKC BioSampler; b, c, f: connecting tubes; d: 3-way stopcock with a tube; e: 5 mm trocar. On the right: SKC Bio Sampler disassembled.

**Figure 2:**
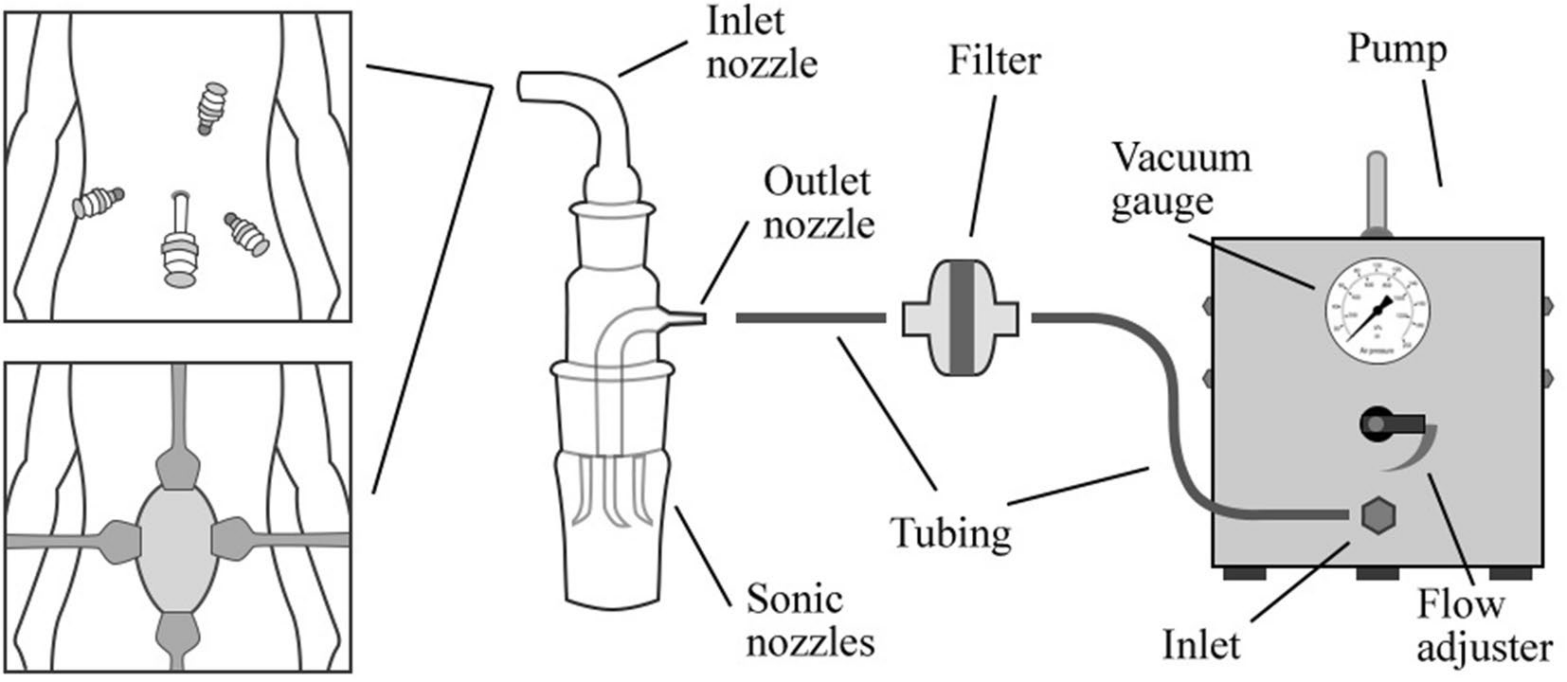
Collecting the surgical smoke with SKC BioSampler in MIS (upper left corner) and open (lower left corner) surgery.

The surgical smoke samples from the pneumoperitoneum during MIS and the approximate surgical field during open surgery and air samples from the OR were collected in 15 ml of sterilized phosphate-buffered saline (PBS) solution using an SKC BioSampler. The SKC BioSampler is a highly efficient gas collection device with a high-volume sonic pump used to analyze airborne microorganisms^17^. It contains one inlet, three sonic, and one outlet nozzles (Fig. 1). The inlet nozzle connects to the 5-mm trocar during MIS directing the surgical smoke into the SKC BioSampler. During open surgery, the inlet nozzle connects to an 8.0 mm wide sterile tube attached to the surgical field and directs the surgical smoke into the SKC BioSampler. The air samples from the OR are collected similarly to open surgery with an 8.0 mm tube connected to the inlet nozzle. The outlet nozzle connects to a vacuum pump at a flow rate of 12.5 L/min and a pressure drop of 15 mmHg. A Clear-Therm™ 3 HMEF filter was inserted between the SKC BioSampler and the vacuum pump to ensure safety (Fig. 2). The SKC BioSampler is then aseptically sealed and transported to the laboratory for analysis. After use, the SKC BioSampler is appropriately sterilized and made available for reuse.

The samples were processed and screened for SARS-CoV-2 RNA in a certified clinical virology laboratory. The RNA was extracted with automated commercial magnetic bead-based procedures using QIASymphony and the DSP Virus/Pathogen Mini Kit (QIAGEN, Hilden, Germany) or chemagic 360 and the Viral DNA/RNA 300 Kit H96 (PerkinElmer, Rodgau, Germany) complying with the manufacturer's instructions. The extracted RNA was tested for SARS-COV-2 RNA with a commercial dual-target (E&N gene) RT-qPCR-Assay (TIB MOLBIOL, Berlin, Germany) employing a Roche LightCycler I or II. Some nasopharyngeal swabs were also tested with a commercial SARS-CoV-2 RT-PCR assay on the fully automated Roche Cobas 6800 instrument (Roche Diagnostics, Germany). The positive surgical smoke samples were to be sent to the appropriate laboratory for growth culture analysis to test for potential infectivity.

## Statistics

Statistical analyses were performed using IBM SPSS Statistics version 24 (IBM Corp, Armonk, New York, United States). The presented data were described using mean values ± standard deviation (SD), median [25th–75th percentile], or frequencies and percentages. Differences between the two groups were evaluated using the chi-square test or Fisher's exact tests for categorical variables and unpaired t-tests or Mann–Whitney U tests for continuous variables, depending on the assumption of normality holds. All reported p values are two-sided, and p values of < 0.05 were considered statistically significant.

## Results

**Figure 3:**
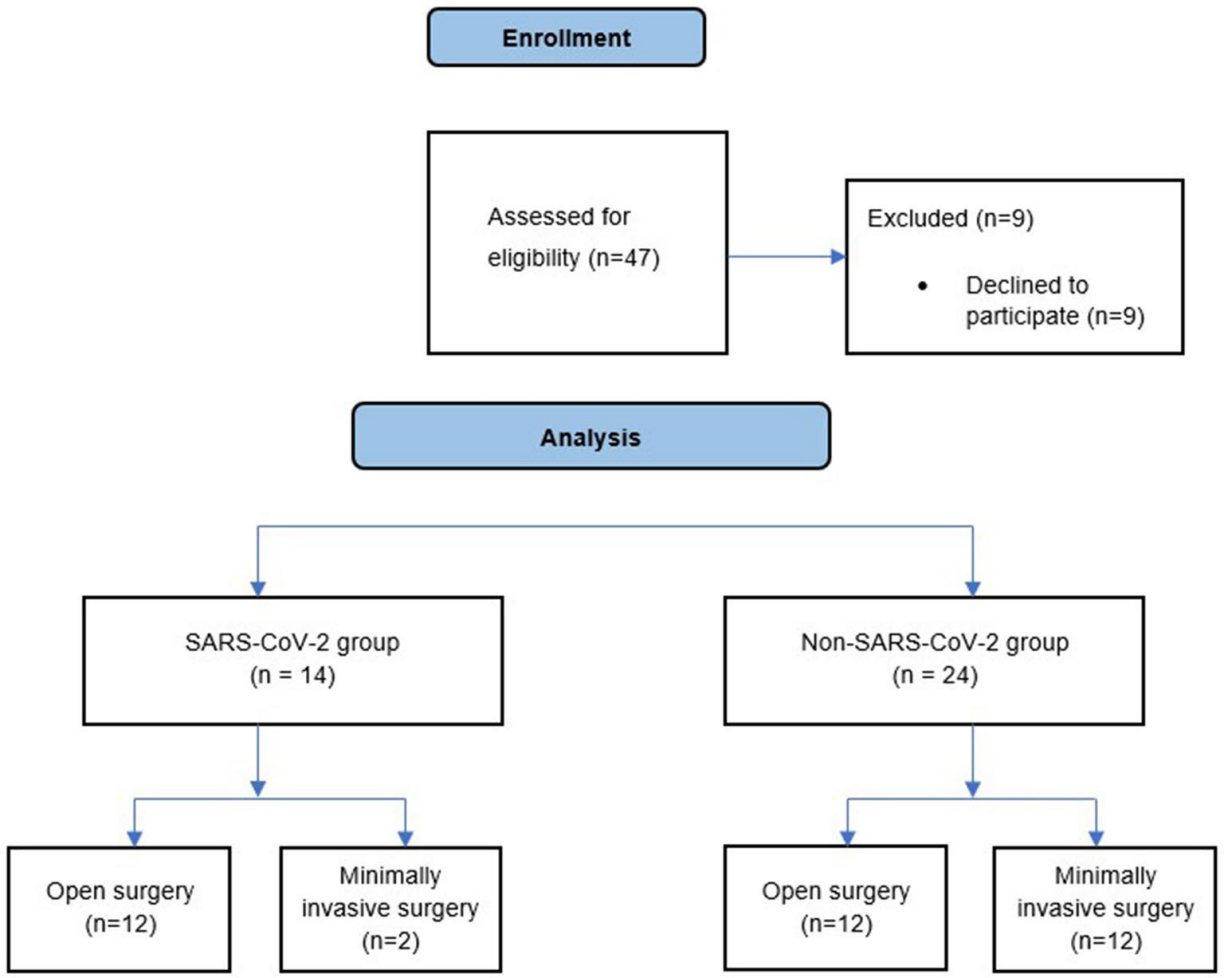
Flow chart of the study design.

A total of 38 patients were assessed for eligibility for the study. Nine patients declined to participate in the study. Fourteen patients with SARS-CoV-2 infection were included in the study: 12 underwent open abdominal surgery, and 2 underwent MIS. The healthy cohort or non-SARS-CoV-2 group included 24 patients: 12 underwent open, and 12 underwent MIS procedure (Fig. 3).

The preoperative parameters of the patients are shown in Table 1. The SARS-CoV-2 group had significantly higher BMI and weight (31.9 ± 9.2 vs. 25.1 ± 5.5, *p* = 0.007, 91.3 kg ± 30.6 kg vs. 74.8 kg ± 18.7 kg, *p* = 0.046, respectively), and more often COPD and DM type II (5 vs. 2, *p* = 0.036, 9 vs. 4 *p* = 0.003, respectively). There were no other differences between the two groups.

**Table 1:** Preoperative parameters of the patients.

Parameter	SARS-CoV-2 group n = 14 (36.8%)	Control group n = 24 (63.2%)	*p* value
Age (years)	57.2 ± 15.7	57.6 ± 10.3	0.931
Female gender	8 (57.1%)	12 (50.0%)	0.671
Height (cm)	168.4 ± 10.4	172.1 ± 8.8	0.253
Weight (kg)	91.3 ± 30.6	74.8 ± 18.7	0.046
BMI (kg/m^2^)	31.9 ± 9.2	25.1 ± 5.5	0.007
CAD	4 (28.6)	3 (12.5)	0.218
AF	1 (7.1%)	1 (4.2%)	0.692
Arterial hypertension	12 (85.7)	14 (58.3)	0.080
COPD	5 (35.7%)	2 (8.3%)	0.036
DM Type II	9 (64.3%)	4 (16.7%)	0.003
CKD	3 (21.4%)	3 (12.5%)	0.467
PAD	1 (7.1%)	2(8.3%)	0.781
Active smoking	4 (28.6%)	8 (33.3%)	0.761

*BMI* Body mass index; *DM* Diabetes mellitus; *CKD* Chronic kidney disease; *PAD* Peripheral arterial disease; *AF* Atrial fibrillation. Data are presented as number (percentage) for categorical variables, mean standard deviation ± for normally distributed or median, and [25th and 75th percentile] for not normally distributed continuous variables. Accordingly, Chi-Quadrat, exact Fisher, Student’s t-test, or Mann–Whitney U test was used to compare.

**Figure 4:**
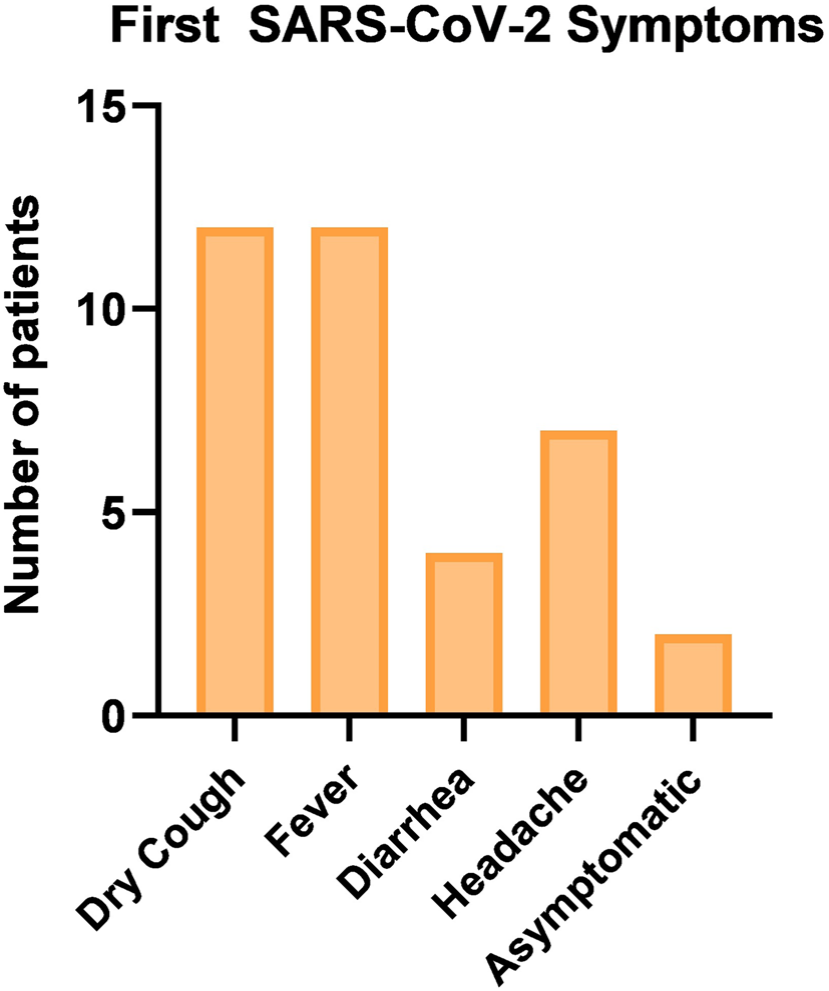
Initial SARS-CoV-2 symptoms of the included patients.

Two patients in the SARS-CoV-2 group had no initial symptoms of infection (Fig. 4). Most of the SARS-CoV-2 patients (n = 12) had the following symptoms: dry cough, fever, and dyspnea. Out of those 12 patients, 4 had diarrhea as one of the initial symptoms in their history.

**Figure 5:**
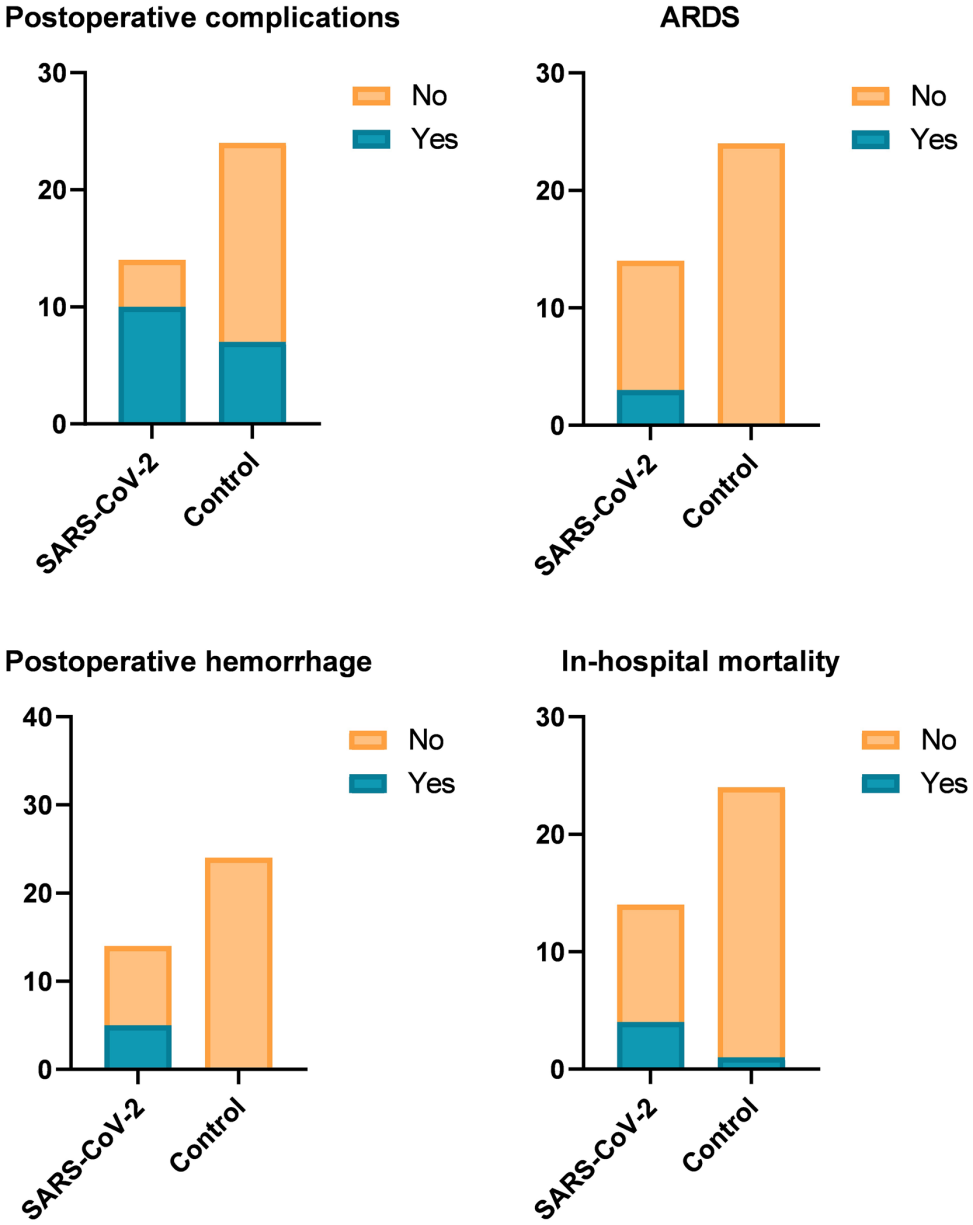
Postoperative outcomes of the patients in SARS-CoV-2 and non-SARS-CoV-2 groups.

The surgical and postoperative parameters of both groups are displayed in Table 2. The postoperative complications were observed significantly more often in SARS-CoV-2 than in the non-SARS-CoV-2 group (10 vs. 7, *p* = 0.011). Postoperative hemorrhage, ARDS, and acute kidney failure were present more frequently in patients with SARS-CoV-2 than those without SARS-CoV-2 infection (5 vs. 0, *p* = 0.002; 3 vs. 0, *p* = 0.018; 3 vs. 0, *p* = 0.018, respectively) (Fig. 5). The major complications (CD ≥ IIIa) were more often observed in SARS-CoV-2 than in the non-SARS-CoV-2 group (10 vs. 4, *p* = 0.001).

**Table 2:** Surgical and postoperative parameters of the patients.

Parameter	SARS-CoV-2 group n = 14 (36.8%)	Control group n = 24 (63.2%)	*p* value
Surgical approach			0.039
Open	12 (85.7%)	12 (50.0%)	
Laparoscopic	2 (14.3%)	12 (50.0%)	
CDC postoperative complications	10 (71.4%)	7 (29.2%)	0.011
I	0 (0.0%)	0 (0.0%)	–
II	0 (0.0%)	3 (12.5%)	0.168
IIIa	1 (7.1%)	2 (8.3%)	0.896
IIIb	2 (14.3%)	2 (8.3%)	0.564
IVa	2 (14.3%)	0 (0.0%)	0.057
IVb	1 (7.1%)	0 (0.0%)	0.185
V	4 (28.6%)	0 (0.0%)	0.006
Reoperation	3 (21.4%)	2 (8.3%)	0.249
Postoperative hemorrhage	5 (35.7%)	0 (0.0%)	0.002
ARDS	3 (21.4%)	0 (0.0%)	0.018
Akute kidney failure	3 (21.4%)	0 (0.0%)	0.018
Pancreatic fistula	0 (0.0%)	2 (8.3%)	0.267
Wound infection	2 (14.3%)	4 (16.7%)	0.846
LOS	31 ± 27.1	21.4 ± 24.7	0.270
In-hospital mortality	4 (28.6%)	1 (4.2%)	0.052

*CDC* Clavien-Dindo classification; *ARDS* Acute respiratory distress syndrome; *LOS* Length of stay. Data are presented as number (percentage) for categorical variables, mean standard deviation ± for normally distributed or median, and [25th and 75th percentile] for not normally distributed continuous variables. Accordingly, Chi-Quadrat, exact Fisher, Student’s t-test, or Mann–Whitney U test was used to compare.

The SARS-CoV-2 RT-PCR analyses are shown in Table 3. Blood, urine, stool, as well as surgical smoke, and OR air samples were SARS-CoV-2 negative in all 14 patients with SARS-CoV-2 infection. One patient had a SARS-CoV-2 positive intraabdominal swab with a Ct value of 36.78. However, there were no viral particles detectable in the sample. Due to the severity of the patient's condition and the necessity of rapid transfer to the intensive care unit, only 7 out of 14 HME filter membranes and 1 out of 14 stool samples were collected and analyzed. Only 1 sample of the HME filter was positive for SARS-CoV-2 viral particles.

**Table 3:** RT-PCR test results in the SARS-CoV-2 group.

Patients	Surgical approach	Nasopharyngeal swab	Ct value	Lineage	HME filter membrane	Intraabdominal swab	Surgical smoke*	Blood	Urine	Stool	OR air sample**
1	Open	Positive	28.94	B.1.1.7	–	Negative	Negative	Negative	Negative	–	Negative
2	Open	Positive	27.68	B.1.1.7	–	Negative	Negative	Negative	Negative	–	Negative
3	Open	Positive	25.40	B.1.1.7	Positive	Negative	Negative	Negative	Negative	–	Negative
4	Open	Positive	24.09	Inconclusive	Negative	Negative	Negative	Negative	Negative	Negative	Negative
5	Open	Positive	32.54	A.27	Negative	Negative	Negative	Negative	Negative	–	Negative
6	Open	Positive	38.14	N501Y	Negative	Negative	Negative	Negative	Negative	–	Negative
7	Open	Positive	32.92	B.1.1.7	Negative	Negative	Negative	Negative	Negative	–	Negative
8	Open	Positive	24.98	AY.46.6	–	Negative	Negative	Negative	Negative	–	Negative
9	Open	Positive	29.67	AY.4	–	Negative	Negative	Negative	Negative	–	Negative
10	Open	Positive	28.73	BA.1	–	Negative	Negative	Negative	Negative	–	Negative
11	Open	Positive	33.54	B.1.617.2	–	Positive	Negative	Negative	Negative	–	Negative
12	Open	Positive	16.1	B.1.617.2	–	Negative	Negative	Negative	Negative	–	Negative
13	Laparoscopic	Positive	36.22	Inconclusive	Negative	Negative	Negative	Negative	Negative	–	Negative
14	Laparoscopic	Negative***	/	/	Negative	Negative	Negative	Negative	Negative	–	Negative

*RT-PCR* Reverse transcription-polymerase chain reaction; *SARS-CoV-2* Severe acute respiratory syndrome coronavirus 2; *Ct* Threshold cycle; *HME* Heath, and moisture exchanger; (–) samples not obtained.

*From either proximate surgical site in open or pneumoperitoneum in minimally invasive surgery.

**Collected at the proximate end of surgery for 30 min.

***Patient included due to positive PCR Test on admission day. All samples have been taken approximately before, during, or after surgery.

**Figure 6:**
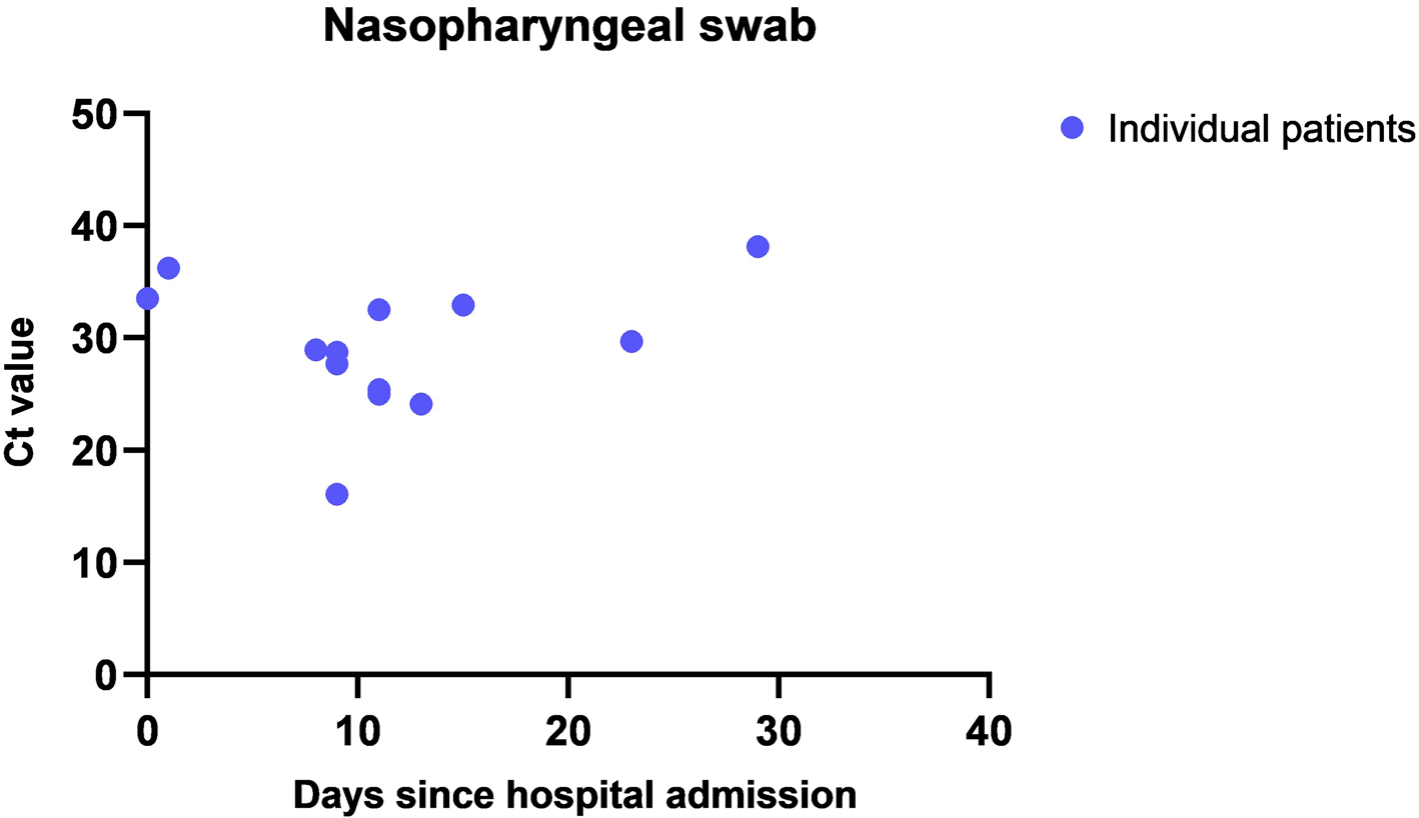
Ct value of the positive nasopharyngeal RT-PCR tests of SARS-CoV-2 patients compared to the day of admission.

**Figure 7:**
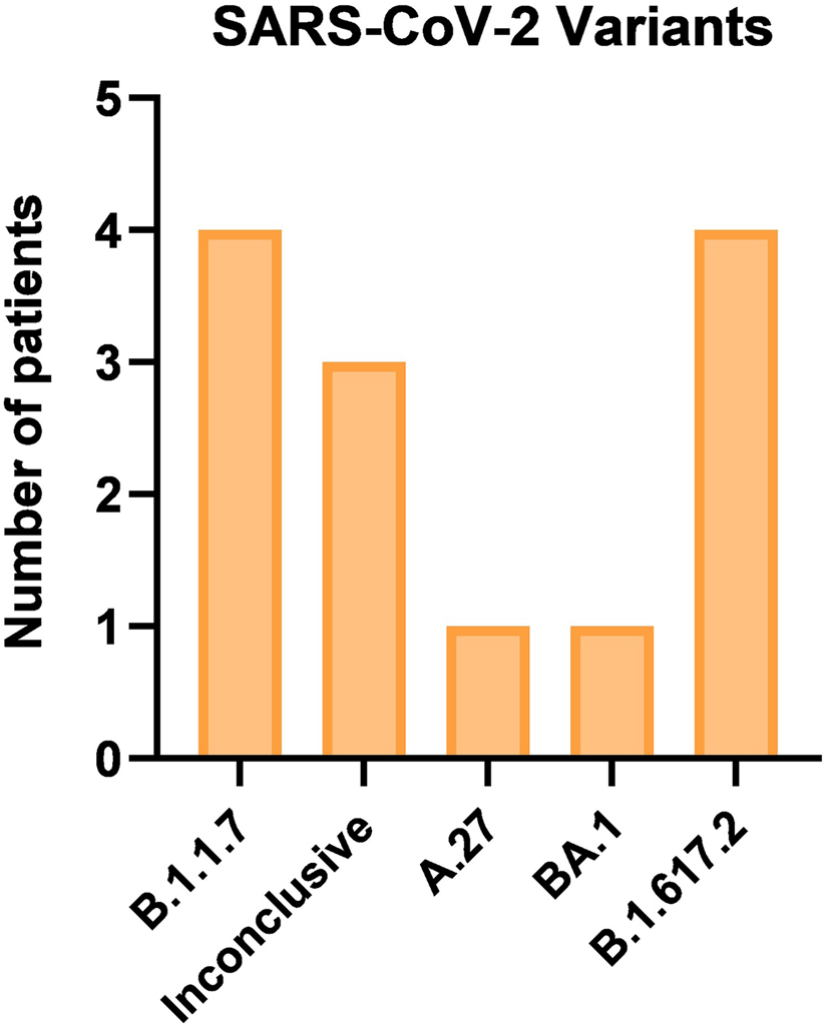
Presentation of the different SARS-CoV-2 lineages detected in RT-PCR tests of the nasopharyngeal swabs.

The Ct values in positive nasopharyngeal swabs differed between 16.10 and 38.14 ranging from 0 to 29 days from admission until the day of surgery (Fig. 6). This indicates further that some patients underwent surgery on the same day (day 0) and others were first treated for their infection symptoms and later developed the complications requiring abdominal surgery (days from 1 until 29). There were five different SARS-CoV-2 lineages in the positive nasopharyngeal swabs of the SARS-CoV-2 patients. The lineage in three patients could not be identified and was termed inconclusive (Fig. 7). All collected samples in 24 non-SARS-CoV-2 patients were SARS-CoV-2 negative.

## Discussion

The present single-center prospective clinical trial assessed the presence of SARS-CoV-2 in surgical smoke in patients with proven SARS-CoV-2 infection undergoing open and MIS procedures. Other samples, such as nasopharyngeal swabs, HME filter membranes, blood, urine, stool samples, and intraabdominal swabs, were also analyzed. Intraoperative nasopharyngeal swabs detected seven different SARS-CoV-2 lineages. SARS-CoV-2 was detected in an intraabdominal swab in one patient, but the presence of SARS-CoV-2 in blood, urine, feces, surgical smoke, or air samples from the OR from the included patients could not be shown.

The COVID-19 pandemic challenged the healthcare systems in the entire world. Due to the detection of SARS-CoV-2 in blood, urine, feces, and organ tissue^19–21,32–35^, concerns have risen about SARS-CoV-2 transmission through aerosol-generating medical procedures such as surgical procedures and endoscopy^11^. In the surgical community, there have been many controversies about the safety of performing non-urgent surgeries in patients with SARS-CoV-2 infection^11,25,36–38^. MIS was especially targeted as a potentially greater risk of SARS-CoV-2 transmission than open surgery due to a higher concentration of smoke in the abdominal cavity^23,25,39,40^. In the further course of time, several studies showed that MIS procedures could provide better control of elimination and produce less surgical smoke than open surgery, given those special filtering mechanisms are implemented^41,42^.

In the present study, the surgical smoke and OR air samples were negative in all 14 SARS-CoV-2 infected patients both in open and MIS procedures. There have been many studies tackling the idea of SARS-CoV-2 aerosolization during open and MIS procedures^30,42–46^, but none of them collected the surgical smoke samples and analyzed them on the presence of SARS-CoV-2 particles. On the other hand, several studies have screened the presence of SARS-CoV-2 during open and MIS in peritoneal fluid, which could be linked to the potential aerosolization of the SARS-CoV-2 particles^47–54^. Both SARS-CoV-2 positive and negative peritoneal fluid samples were reported, which led to inconclusive statements about SARS-CoV-2 transmission through surgical smoke. However, many urgent surgical procedures are standardized with MIS, such as cholecystectomy and appendectomy. While smoke evacuation and filtration systems can be used during open and MIS procedures, MIS offers the unique advantage of being able to almost entirely contain the surgical smoke in the abdominal cavity. In combination with tightly fitting laparoscopic ports, an evacuation system can be used to minimize the release of the potential airborne virus into the OR environment while simultaneously evacuating the surgical smoke. This contrasts with the smoke evacuation during open surgical procedures, where containment of the surgical smoke is challenging, if not impossible, if the goal is to evacuate the surgical smoke completely.

In the current literature, three studies report on SARS-CoV-2 in surgical smoke. One of those studies collected surgical smoke in one patient during MIS appendectomy^55^. Although the surgical smoke samples were negative, the sampling methodology could not be verified since the authors used an improvised 3-way circuit with a stop-valve on the trocar site attached to a syringe with 20 ml saline solution. Furthermore, the surgical smoke was collected three times during the procedure without mentioning how long each collecting phase took. The second study concluded that SARS-CoV-2 is present in surgical smoke by detecting SARS-CoV-2 in 2 out of 17 patients undergoing MIS from a trocar valve filter^30^. One of the two patients had a positive filter sample (Ct value 42.75) without a concomitant positive nasopharyngeal sample. The authors did not address this discrepancy in results or test the infectivity of the positive trocar filter samples. The third study by Haddadin et al.^56^ reported, similarly to the first study, the collection of surgical smoke using an improvised sampling tube with a fluid collector attached to the trocar site in 11 patients undergoing MIS without detecting SARS-CoV-2 in the samples.

Yokoe et al. have conducted a model experiment to evaluate the possibility of lowering the transmission risk of SARS-CoV-2 by filtration through a surgical mask. The authors used a vacuum system to obtain surgical smoke as a hydrosol from infected HeLa-ACE2-TMPRSS2 cells with human coronavirus 229E (HCoV-229) incised by an electric and ultrasonic scalpel. They found viral RNA within the induced surgical smoke but without proven infectivity. It was hypothesized that treating the cell cultures with surgical devices damages the viral particles, resulting in loss of infectivity^57^. On the other hand, the HcoV-229 might have similar stability as SARS-CoV-2, but it is phylogenetically distinct from it, which could lead to poor transferability of the study to the SARS-CoV-2 in-vivo infected cells^58^. Furthermore, there is the clinical aspect of the induction of surgical smoke from cells and tissues which have not been primarily and intentionally infected with SARS-CoV-2 and therefore contain potentially fewer viral particles. Consequently, it could be that SARS-CoV-2 was not detected in surgical smoke during open and MIS procedures due to the low number of SARS-CoV-2 viral particles in the tissue itself.

The present study collected air samples from the OR approximately toward the end of the surgery in SARS-CoV-2 infected patients undergoing open and MIS procedures. All 14 air samples from the OR did not contain SARS-CoV-2 viral particles. Barberá-Riera et al. tested samples collected for three weeks inside two ORs for 24 h at 38 L/min in a quartz filter. The authors tested the mentioned aerosol samples and three laparoscopy filters on SARS-CoV-2 genetic material. Despite low concentration, they reported that 11.3% of aerosol samples were positive for SARS-CoV-2 genetic material. However, the three laparoscopy filters were SARS-CoV-2 negative, raising the question of the source of air contamination in the ORs. The authors speculated that aerosol-generating medical procedures during open surgery could be a potential source of air contamination. However, another source could also be healthcare workers with asymptomatic SARS-CoV-2 infection^59^. The present study did not show SARS-CoV-2 presence in the OR air samples during open and MIS procedures nor in surgical smoke. Therefore, it is not likely that the source of air contamination came from aerosol-generating medical procedures during open surgery. Instead, the contamination could easily appear through aerosol-generating medical procedures such as intubation or asymptomatic healthcare workers transmission.

This study has detected one SARS-CoV-2 positive intraperitoneal swab. The patient had an acute colon perforation and underwent urgent open abdominal surgery. Since several studies reported SARS-CoV-2 in fecal samples^20–22,33^, it could be that the SARS-CoV-2 contaminated the abdominal cavity through colon perforation. However, there has also been a report of two SARS-CoV-2 positive peritoneal fluid samples in patients undergoing urgent abdominal surgery without visceral perforation^53,54^. On the other hand, Jakimiuk et al.^60^ tested peritoneal fluid samples in 34 SARS-CoV-2 patients undergoing cesarian section, out of which all 34 peritoneal fluid samples were SARS-CoV-2 negative. Flemming et al.^61^ could not prove the presence of SARS-CoV-2 in peritoneal fluid in a SARS-CoV-2-infected patient undergoing emergency MIS cholecystectomy. None of the studies conducted SARS-CoV-2 infectivity testing in the mentioned positive samples.

Four SARS-CoV-2 patients had diarrhea as one of their first symptoms. The increased expression of the ACE2 receptors has been detected in the gastrointestinal tissue of patients with gastrointestinal symptoms during the SARS-CoV-2 infection^62,63^. ACE2 expression on surface cells of the gastrointestinal tract could mediate the invasion and amplification of the virus, causing gastrointestinal symptoms. This could explain the presence of the virus in patients’ stool samples reported by some studies^64^. Although the high expression of ACE2 receptors in the gastrointestinal tract is probably the cause of SARS-CoV-2 gastrointestinal symptoms, it is still uncertain how it affects the viral spread in abdominal surgeries.

The study's main limitations are the small sample size, especially within the MIS subgroup, and the single-center nature of the study. The small sample size was due to the indications for surgical treatment of the SARS-CoV-2 infected patients and the difficulty of including patients in a multi-centric setting during the COVID-19 pandemic. Another important aspect observed in the study is the high Ct value of the SARS-CoV-2 patients at the time of surgery, which could potentially affect outcomes. The SARS-CoV-2 group of the study underwent mostly emergency surgeries due to complications that occurred in a rather late SARS-CoV-2 infection phase secondary to SARS-CoV-2 and sepsis-related coagulation disorders^65–69^. It would be interesting to examine samples collected during the high-infectivity phase in patients with low Ct values and compare them with the current outcomes. Furthermore, only 4 patients in the SARS-CoV-2 group had gastrointestinal symptoms (diarrhea), which could potentially influence the detection of the virus in the samples during the abdominal surgeries (Fig. 4).

Another limiting factor of the study is the one SARS-CoV-2 negative nasopharyngeal PCR test in the SARS-CoV-2 group sampled during the surgery. The patient was included in the study according to the SARS-CoV-2 positive nasopharyngeal PCR test on admission day, two days before the surgery. This could be due to the low infectivity of the patient at the time of the surgery, which could affect the study's outcomes. Another potential reason for the negative PCR test could be insufficient sampling quality despite professionally trained staff.

This study showed no detectable viral particles of SARS-CoV-2 in surgical smoke sampled during MIS and open surgery. Thus, the discussed risk of transmission of SARS-CoV-2 via surgical smoke could not be confirmed in the present study. However, given the paucity of data, taking precautions in the OR is reasonable since viral particles can be aerosolized during intubation and extubation. It is possible that MIS offers the opportunity for better containment and filtration of the surgical smoke compared with laparotomy and continues to offer health benefits, particularly during the current pandemic when hospital resources as well as minimizing viral exposure risks are so critical to moderating this public health crisis^70^.

## Data Availability

All data generated or analysed during this study are included in this published article.
